# Shortened duration whole body ^18^F-FDG PET Patlak imaging on the Biograph Vision Quadra PET/CT using a population-averaged input function

**DOI:** 10.1186/s40658-022-00504-9

**Published:** 2022-10-29

**Authors:** Joyce van Sluis, Johannes H. van Snick, Adrienne H. Brouwers, Walter Noordzij, Rudi A. J. O. Dierckx, Ronald J. H. Borra, Adriaan A. Lammertsma, Andor W. J. M. Glaudemans, Riemer H. J. A. Slart, Maqsood Yaqub, Charalampos Tsoumpas, Ronald Boellaard

**Affiliations:** 1 Department of Nuclear Medicine and Molecular Imaging, University Medical Center Groningen, University of Groningen, Hanzeplein 1, 9713GZ Groningen, The Netherlands; 2 Department of Radiology and Nuclear Medicine, Amsterdam University Medical Centers, Location VUMC, Amsterdam, The Netherlands

**Keywords:** Patlak, Dynamic imaging, Vision Quadra, PET/CT, Scan duration

## Abstract

**Background:**

Excellent performance characteristics of the Vision Quadra PET/CT, e.g. a substantial increase in sensitivity, allow for precise measurements of image-derived input functions (IDIF) and tissue time activity curves. Previously we have proposed a method for a reduced 30 min (as opposed to 60 min) whole body ^18^F-FDG Patlak PET imaging procedure using a previously published population-averaged input function (PIF) scaled to IDIF values at 30–60 min post-injection (p.i.). The aim of the present study was to apply this method using the Vision Quadra PET/CT, including the use of a PIF to allow for shortened scan durations.

**Methods:**

Twelve patients with suspected lung malignancy were included and received a weight-based injection of ^18^F-FDG. Patients underwent a 65-min dynamic PET acquisition which were reconstructed using European Association of Nuclear Medicine Research Ltd. (EARL) standards 2 reconstruction settings. A volume of interest (VOI) was placed in the ascending aorta (AA) to obtain the IDIF. An external PIF was scaled to IDIF values at 30–60, 40–60, and 50–60 min p.i., respectively, and parametric ^18^F-FDG influx rate constant (*K*_*i*_) images were generated using a t* of 30, 40 or 50 min, respectively. Herein, tumour lesions as well as healthy tissues, i.e. liver, muscle tissue, spleen and grey matter, were segmented.

**Results:**

Good agreement between the IDIF and corresponding PIF scaled to 30–60 min p.i. and 40–60 min p.i. was obtained with 7.38% deviation in *K*_*i*_. Bland–Altman plots showed excellent agreement in *K*_*i*_ obtained using the PIF scaled to the IDIF at 30–60 min p.i. and at 40–60 min p.i. as all data points were within the limits of agreement (LOA) (− 0.004–0.002*,* bias: − 0.001); for the 50–60 min p.i. *K*_*i*_, all except one data point fell in between the LOA (− 0.021–0.012, bias: − 0.005).

**Conclusions:**

Parametric whole body ^18^F-FDG Patlak *K*_*i*_ images can be generated non-invasively on a Vision Quadra PET/CT system. In addition, using a scaled PIF allows for a substantial (factor 2 to 3) reduction in scan time without substantial loss of accuracy (7.38% bias) and precision (image quality and noise interference).

**Supplementary Information:**

The online version contains supplementary material available at 10.1186/s40658-022-00504-9.

## Introduction

Non-invasive positron emission tomography integrated with computed tomography (PET/CT) imaging using ^18^F-2-fluoro-2-deoxy-D-glucose (^18^F-FDG) provides both metabolic and anatomic information [1] and is widely used in oncology [2–4] and many other clinical indications. In oncology, PET/CT has become part of the daily clinical routine including initial diagnosis, staging, prognosis, radiation therapy planning, and monitoring response to treatment [4–6].

The semi-quantitative standardised uptake value (SUV) of ^18^F-FDG, derived from static images obtained at 60 min post-injection (p.i.), is most commonly used as a surrogate of metabolic activity for tumour uptake quantification [5]. Following standardisation methods such as the European Association of Nuclear Medicine (EANM) procedure guidelines for tumour imaging regarding patient preparation, PET image acquisition and reconstruction settings, and analysis methods can mitigate SUV variability to a great extent [5, 7, 8]. However, these standardisation methods are not able to account for changes in plasma kinetics, due to, for example, treatment, and no distinction between specific and nonspecific uptake possibly causing a dissociation between inaccurate SUV measurements and actual tumour metabolic activity [9–11].

In contrast, dynamic PET imaging, both single-bed or whole body dynamic (e.g. via multi-bed or continuous bed motion modes) [12, 13], is able to include this information as it allows spatiotemporal activity concentration measurement, providing voxel-wise metabolic information after applying full kinetic- or Patlak analyses [13–15].

Fully quantitative whole body parametric PET imaging for non-invasive quantification of ^18^F-FDG kinetics throughout the body is now possible using a long axial field of view (LAFOV) Siemens Biograph Vision Quadra PET/CT system (Siemens Healthineers, Knoxville, TN, USA) (hereinafter referred to as Vision Quadra). Its 106 cm long axial coverage captures the heart and all other organs of interest, including possible tumour lesions, simultaneously and continuously within a single bed position. However, the large axial field of view (FOV) (including the CT part) hampers easy access to the (radial) blood vessels to perform blood sampling, the gold standard to obtain the plasma input curve. The excellent performance characteristics of the Vision Quadra, such as a substantial increase in sensitivity [16], allows for precise measurements of image-derived input functions (IDIF) and tissue time activity curves (TAC). However, in order to generate whole body parametric PET images, the slow kinetics of ^18^F-FDG require scanning for at least 45–60 min [17]. This longer scan duration reduces patient throughput and comfort, whereas improvements in PET technology [3] and the large axial FOV actually enable acquisition of whole body static images at substantially reduced scan durations, thereby providing the potential of increasing patient throughput.

The use of a population-averaged input function (PIF) could obviate the need to acquire ^18^F-FDG tracer distribution information immediately following injection, thereby minimizing whole body dynamic imaging to an interval of 30–60 min p.i. [18–22]. Moreover, in the case of sufficient increase in signal-to-noise ratio achieved with the Vision Quadra, Patlak analyses could provide accurate voxel-wise metabolic information with fewer dynamic time frames. The feasibility of the current approach as previously described by van Sluis et al. [23] has been investigated in [22] for the 2 m LAFOV uEXPLORER PET/CT system (United Imaging Healthcare, Houston, TX, USA), with a sensitivity of 174 kcps/MBq measured at the centre of the FOV [24], but has not yet been explored for the 106 cm Vision Quadra, measuring a sensitivity of 82.6 kcps/MBq in the centre of the FOV [16]. In our current study, we aimed to assess if the lower sensitivity of the Vision Quadra compared to the uEXPLORER would still allow to perform shortened Patlak imaging with sufficient image quality and quantitative performance. Other described methods for reduced scan duration whole body Patlak imaging, investigated for the uEXPLORER and Vision Quadra, include dual time point dynamic acquisition [25, 26]. Our proposed method is based on a single time point shortened dynamic scan, which might be a more practical approach regarding patient scheduling in a busy clinic.

Previously, data simulations were performed to evaluate a method for reduced 30 min whole body ^18^F-FDG Patlak PET imaging using an external PIF, obtained by arterial blood sampling, scaled to IDIF values at 30–60 min p.i. [23]; the PIF was obtained during baseline scans, prior to any treatment, in a comparable patient cohort. The aim of the present study was to apply this method using clinically acquired patient data on the Vision Quadra. A secondary aim was to assess whether even shorter scan time intervals could be used given the high sensitivity of the Vision Quadra system.

## Materials and methods

Oncology patients with suspected lung malignancy (lung cancer or lung metastases), referred for clinical diagnosis, were included in this study. The local Medical Ethics Review Committee of the University Medical Center Groningen waived the need for formal ethical review (waiver number METc2020/554). Following the standardised procedure guidelines for tumour imaging, patients were instructed to fast for at least 6 h prior to ^18^F-FDG injection and blood glucose levels were no higher than 8.3 mmol/L. All patients received a standard weight-based (3 MBq/kg) injection of ^18^F-FDG.

Scans were acquired on a Vision Quadra PET/CT [16]. Each patient underwent a low dose CT from the vertex to the mid thighs (X-ray tube current 35 mAs, tube voltage of 100 kV, and spiral pitch factor of 1.1) which was used for both anatomical information and PET attenuation correction. After a delay of 10 s following automated ^18^F-FDG bolus injection using a MEDRAD Intego PET infusion system (Bayer Pharmaceuticals, Berlin, Germany) and a saline flush of 30 mL, 65 min listmode dynamic PET data were acquired. Data were binned over 31 frames using the following frame durations: 6 × 10, 3 × 20, 6 × 30, 5 × 60, and 11 × 300 s. In addition, the last 10 min dynamic frames were summed to obtain a conventional static scan at 60 min p.i.

Dynamic PET images were reconstructed using European Association of Nuclear Medicine Research Ltd. (EARL) standards 2 reconstruction settings to obtain data that complied with European guidelines for multicentre PET image quantification and harmonisation [27]. These EARL standards 2 reconstruction settings were a three-dimensional (3D) ordered-subset expectation maximisation (OSEM) algorithm with 4 iterations, 5 subsets and a matrix size of 220 × 220 × 708 with a voxel size of 3.3 × 3.3 × 1.5 mm^3^, time of flight (ToF), resolution modelling (PSF), and a 5 mm full width at half maximum Gaussian filter. PET data were corrected for random, scatter, attenuation, and radioactive decay.

For each dynamic PET dataset, a volume of interest (VOI) was placed in the ascending aorta (AA) [28] to derive an IDIF using in-house developed software. The VOI in the AA consisted of 2 × 2 voxels in 6 axial slices in the lumen of the large artery. To determine the ^18^F-FDG influx rate constant (*K*_*i*_) and the total blood distribution volume $V$, the measured TAC and input function (IF) serve as input for a voxel-wise Patlak analysis according to Eq. 1 [29]:

1 $$\frac{{C(t_{n} )}}{{C_{{\text{P}}} \left( {t_{n} } \right)}} = K_{i} \frac{{\mathop \int \nolimits_{0}^{{t_{n} }} C_{P} \left( \tau \right){\text{d}}\tau }}{{C_{P} \left( {t_{n} } \right)}} + V, t_{n} > t*,\quad n = 1 \ldots N$$

where $C\left( t \right)$ is the measured TAC at each voxel, $C_{{\text{P}}} \left( t \right)$ the IDIF or PIF, and $t_{n}$ with $n = 1 \ldots N$ representing the mid-time points for the $N$ dynamic PET frames. For the calculations, a $t$* of 30, 40 or 50 min was used as the time after which relative kinetic equilibrium between blood and reversible compartment is assumed, i.e. when the Patlak plot becomes linear. Subsequently, a previously published PIF [30], see Additional file 1: Fig. S1, was scaled to IDIF values at 30–60, at 40–60, and at 50–60 min p.i., respectively, corresponding with the t* values mentioned above. This resulted in 4 IFs per dataset: one IDIF obtained at the scan time interval from 0 to 60 min (IDIF), one scaled PIF to the IDIF at the scan time interval from 30 to 60 min (PIF_30), one PIF scaled to the IDIF at the scan time interval from 40 to 60 min (PIF_40), and one PIF scaled to the IDIF at the scan time interval from 50 to 60 min (PIF_50). For each IF, parametric ^18^F-FDG *K*_*i*_ images were generated. In these *K*_*i*_ images, tumour lesions were segmented using a semi-automated segmentation method (50% of SUV_peak_ isocontour) using the in-house developed software tool quAntitative onCology moleCUlaR Analysis suiTE (ACCURATE) [31]. In addition, the semi-automated segmentation method was also used to segment grey matter, a 3-cm-diameter spherical VOI was placed in the liver and 2-cm-diameter spherical VOIs were placed in the upper thigh muscle and spleen to assess *K*_*i*_ in healthy organs with clearly different *K*_*i*_ values.

Agreement between lesion *K*_*i*_ obtained using the IDIF and corresponding PIF scaled at 30–60, 40–60, and at 50–60 min p.i., respectively, was assessed using Bland–Altman plots. The coefficient of determination (*R*^2^) was obtained to explore the extent to which variability in lesion *K*_*i*_ obtained using the scaled PIFs can be related to variability in the IDIF. Bland–Altman plots were also used to assess agreement between normal tissue *K*_*i*_ obtained using the IDIF and the various scaled PIFs.

To assess the effect of scan duration on noise levels, the standard deviation (SD) in *K*_*i*_ derived from the liver VOI using the IDIF and PIF scaled to the IDIF at 30–60 min p.i. was compared with *K*_*i*_ obtained using the PIF scaled to the IDIF at various shortened scan time intervals. Moreover, healthy tissue *K*_*i*_ measured in liver, muscle tissue, spleen, and grey matter obtained using the IDIF versus the corresponding scaled PIF at various shortened scan time intervals p.i. were compared using boxplots and ANOVA repeated measures. These analyses were performed using SPSS Statistics, version 27.0 (IBM corp., Armonk, NY, USA).

## Results

**Fig. 1:**
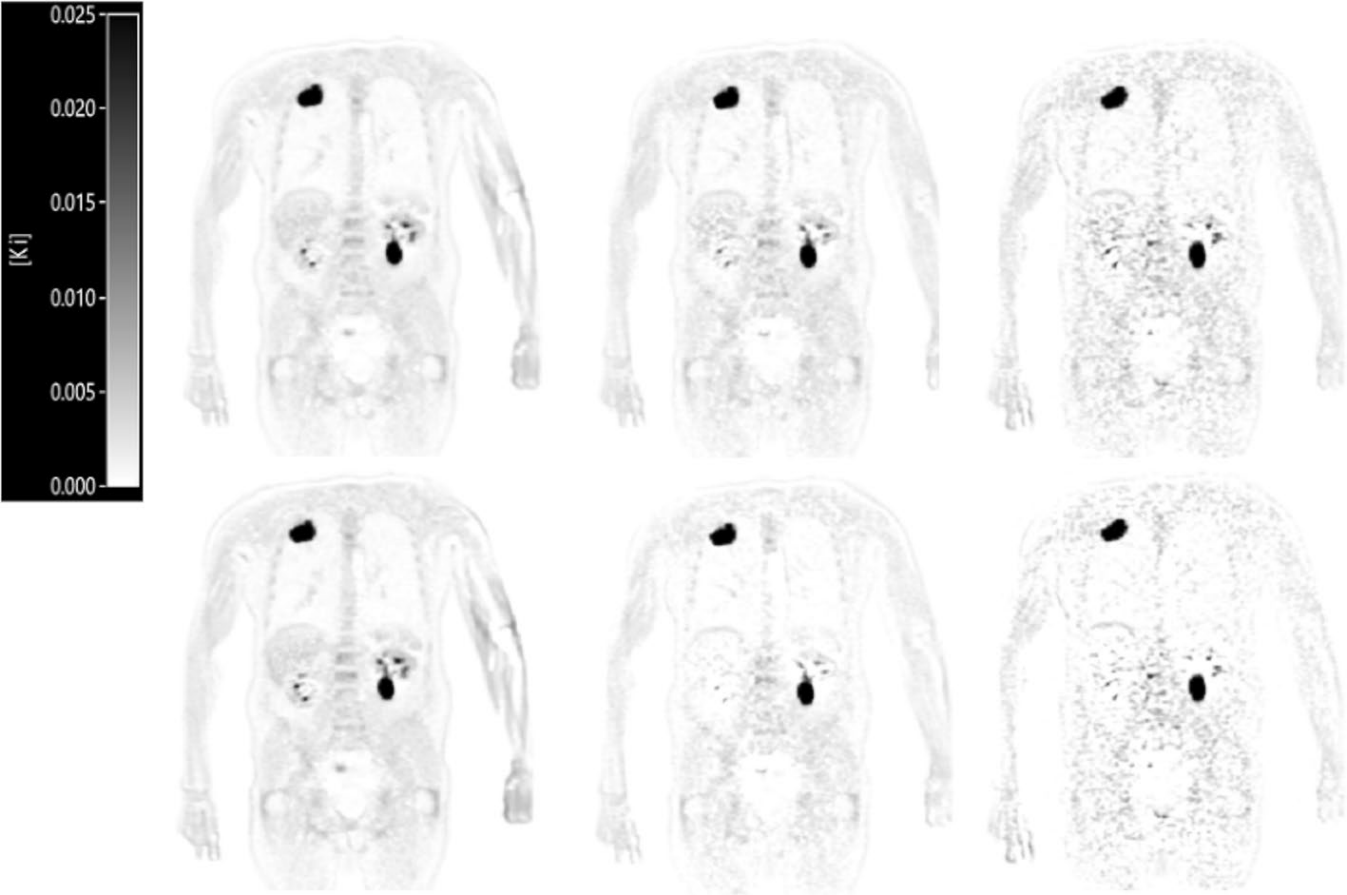
Example *K*_*i*_ images obtained using the IDIF from the AA (top row) and scaled PIF (bottom row). From left to right *K*_*i*_ images are shown that were generated using 30–60, 40–60, and 50–60 min uptake time intervals p.i., respectively. The same interval was used for scaling the population-averaged curve as to simulate actual shortened PET acquisitions of the same intervals

**Fig. 2:**
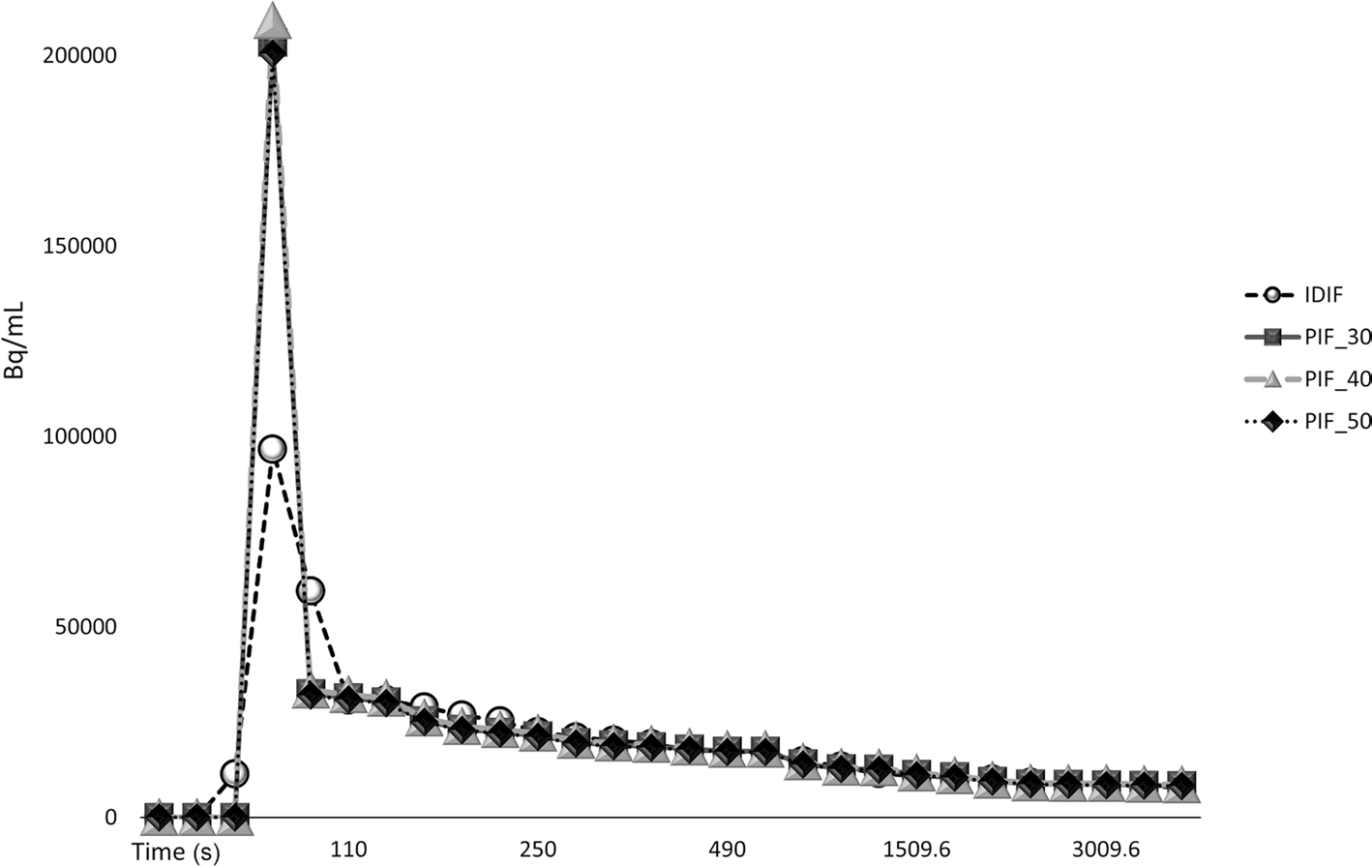
Example IDIF and the PIF scaled towards the example IDIF at various shortened scan time intervals p.i

In total, 12 oncology patients (8 men, 4 women; age 71 ± 4 y [mean ± SD]) with suspected lung malignancy referred for a clinical ^18^F-FDG PET acquisition for initial diagnostic purposes were included in this study. Patients received a standard weight-based (3 MBq/kg) ^18^F-FDG injected activity (weight: 90.2 ± 22.1 kg [mean ± SD]; activity: 272.0 ± 73.3 MBq [mean ± SD]). A total of 20 tumour lesions were segmented and the obtained results are described below. Patient demographics and specific injection parameters can be found in Table 1. Example *K*_*i*_ images illustrating image quality obtained using both the measured IDIF and the scaled PIF at various shortened scan time intervals p.i. are shown in Fig. 1. Figure 2 shows an example IDIF and the PIF scaled towards the example IDIF at various shortened scan time intervals p.i.

**Table 1:** Patient demographics and activity values

Pt	Disease	Sex (M/F)	Age (years)	Weight (kg)	Activity @ scan start (MBq)	Glucose level (mmol/L)
1	Lung cancer	M	64	134	402.3	6.2
2	Lung cancer	M	76	69	208.6	6.0
3	Lung cancer	F	73	89	268.0	5.3
4	Lung cancer	F	68	69	211.0	6.2
5	Lung cancer	M	70	80	245.5	6.7
6	Lung cancer	M	72	85	241.4	5.8
7	Lung cancer	M	75	74	206.1	6.5
8	Lung cancer	M	68	136	443.4	6.8
9	Diffuse large B cell lymphoma	F	80	89	256.0	6.4
10	Lung cancer	M	69	100	308.0	6.0
11	Diffuse large B cell lymphoma	M	65	70	221.9	4.9
12	Lung cancer	F	72	87	251.9	5.9

**Fig. 3:**
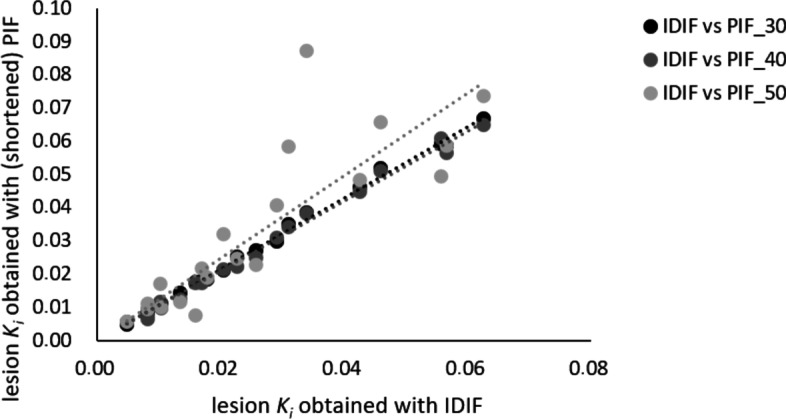
Scatter plots of lesion *K*_*i*_ obtained using the IDIF versus lesion *K*_*i*_ obtained using the PIF scaled to the IDIF at 30–60 min p.i. (PIF _30) and the PIF scaled to the IDIF at shortened scan time intervals p.i. (PIF _40 and PIF _50). Dashed lines represent linear regression fits (with intercept set to (0.0)

Figure 3 illustrates the bias in *K*_*i*_ that is found when using the PIF scaled to the IDIF at scan time intervals p.i. of 30–60 min, 40–60 min, and 50–60 min. The slopes of the linear regression fits, with intercept set to 0.0, indicate that using a PIF overestimates *K*_*i*_ in all cases. Linear regression slope equations for scan time intervals p.i. of 30–60 min, 40–60 min, and 50–60 min: *y* = 1.0738*x*, *y* = 1.0518*x*, and *y* = 1.2405*x*. More specifically, scaling the PIF onto the IDIF at 30–60 min p.i. induces a slight bias in *K*_*i*_ (7.38%). At the shortened scan time interval of 40–60 min p.i., scaling of the PIF onto the IDIF reduces this *K*_*i*_ bias to 5.18%. In cases of scaling the PIF onto the IDIF at the shortest scan time interval of 50–60 min p.i., bias in *K*_*i*_ increases to $>$ 24%. The coefficients of determination (*R*^2^) between the IDIF and the corresponding scaled PIF at 30–60, at 40–60, and at 50–60 min p.i. were 0.999, 0.998, and 0.901, respectively.

**Fig. 4:**
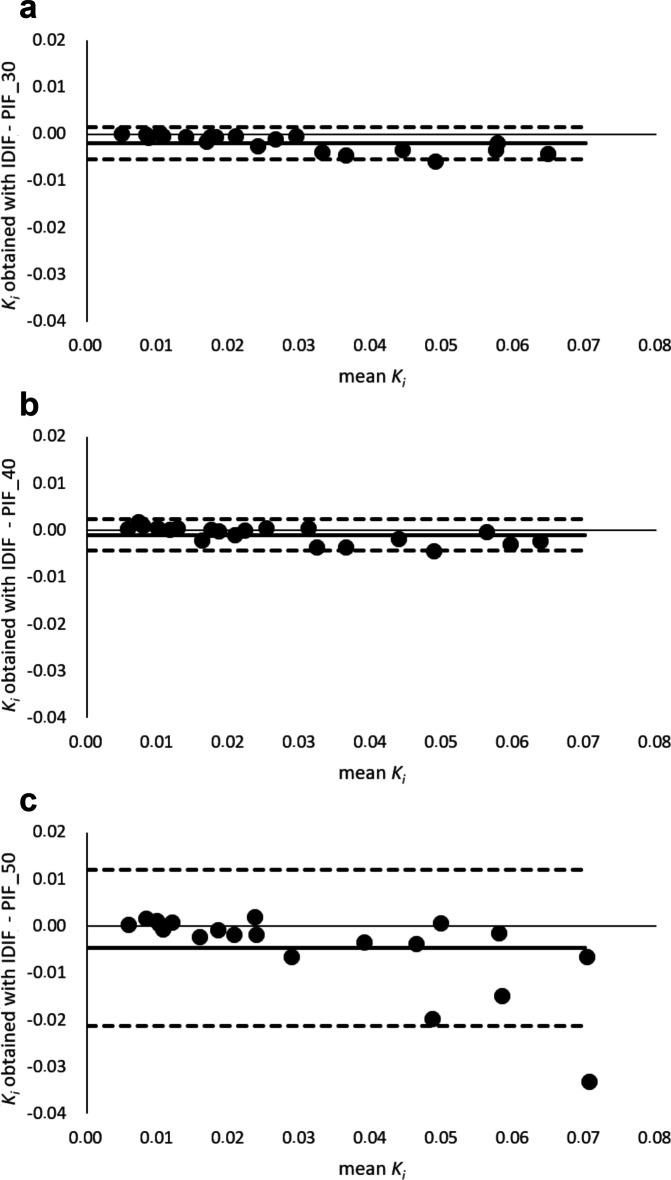
Bland–Altman plot of lesion *K*_*i*_ differences obtained with the IDIF minus the PIF scaled to the scan time interval at 30–60 min p.i. (PIF_30) (**a**), and the PIF scaled to the IDIF at shorter scan time intervals p.i. (PIF_40 and PIF_50) (**b**, **c**). To illustrate differences between the comparisons, the scales on the vertical axes are matched in the subfigures. For readability purposes, these Bland–Altman plots with modified axes are shown in Additional file 1: Fig. S2

Bland–Altman plots (see Fig. 4 and Additional file 1: Fig. S2) to assess the agreement between *K*_*i*_ obtained using the IDIF and the PIF scaled to 30–60 min p.i. showed lower and upper LOA of − 0.005–0.002 and a bias of − 0.002. For the agreement between *K*_*i*_ obtained using the IDIF and the PIF scaled to 40–60 min p.i., upper and lower LOA were − 0.004–0.002 and the bias was − 0.001. When scaling the PIF to the IDIF at the shortest scan time interval of 50–60 min p.i., upper and lower LOA were − 0.021–0.012 and the bias was − 0.005. When scaling the PIF to the IDIF at 30–60 min p.i. as well as 40–60 min p.i., all 20 lesion data points were located in between the LOAs. With regard to scaling of the PIF towards IDIF at the 50–60 min p.i. scan time interval, all but one data point were located in between the LOAs. With regard to assessment of agreement between obtained healthy tissue *K*_*i*_ using the IDIF and PIF scaled to various shortened scan time intervals p.i., Bland–Altman analysis results are summarised in Table 2.

**Table 2:** Healthy tissue *K*_*i*_ Bland–Altman analysis results

Tissue	Comparison	Lower LOA	Upper LOA	Bias	Data points between LOAs (*N* = 12)
Liver	IDIF-PIF_30	− 0.0014	0.0020	0.0003	11
	IDIF-PIF_40	0.0006	0.0048	0.0027	11
	IDIF-PIF_50	0.0021	0.0065	0.0043	12
Muscle	IDIF-PIF_30	− 0.0005	0.0005	− 0.0000	11
	IDIF-PIF_40	− 0.0001	0.0011	0.0005	12
	IDIF-PIF_50	− 0.0004	0.0018	0.0007	12
Spleen	IDIF-PIF_30	− 0.0008	0.0010	0.0001	11
	IDIF-PIF_40	0.0004	0.0034	0.0019	12
	IDIF-PIF_50	0.0011	0.0044	0.0028	12
Grey matter	IDIF-PIF_30	− 0.0026	0.0002	− 0.0012	11
	IDIF-PIF_40	− 0.0087	0.0060	− 0.0013	11
	IDIF-PIF_50	− 0.0073	0.0038	− 0.0018	10

LOA, limit of agreement

**Fig. 5:**
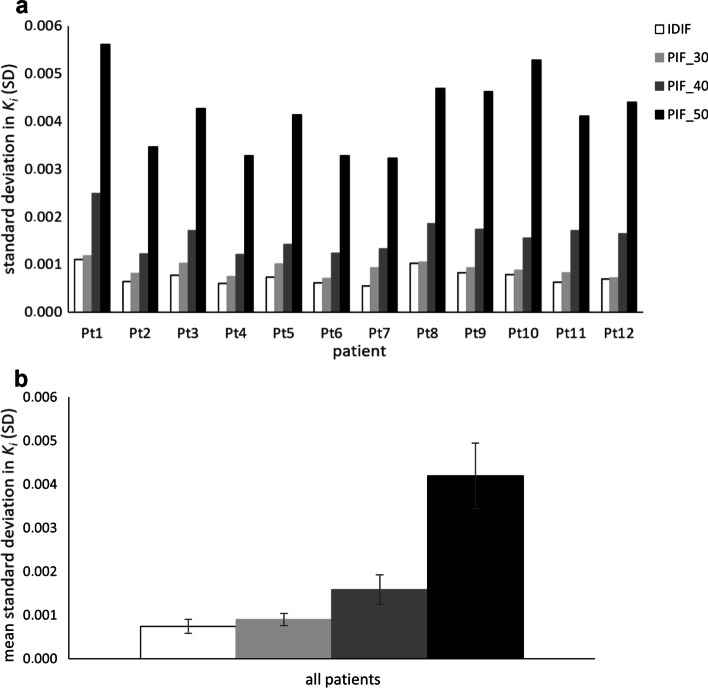
Bar plots of K_i_ SD obtained from the liver VOI (as indication for Patlak image noise) per patient (**a**) and for all patients (**b**) generated with IDIF and various PIF at shorter scan time intervals p.i

In Fig. 5, SD in *K*_*i*_ derived from the liver VOI generated using the IDIF and scaled PIF at various shorter scan time intervals p.i. are shown to indicate the increase in noise levels as scan time is reduced.

**Fig. 6:**
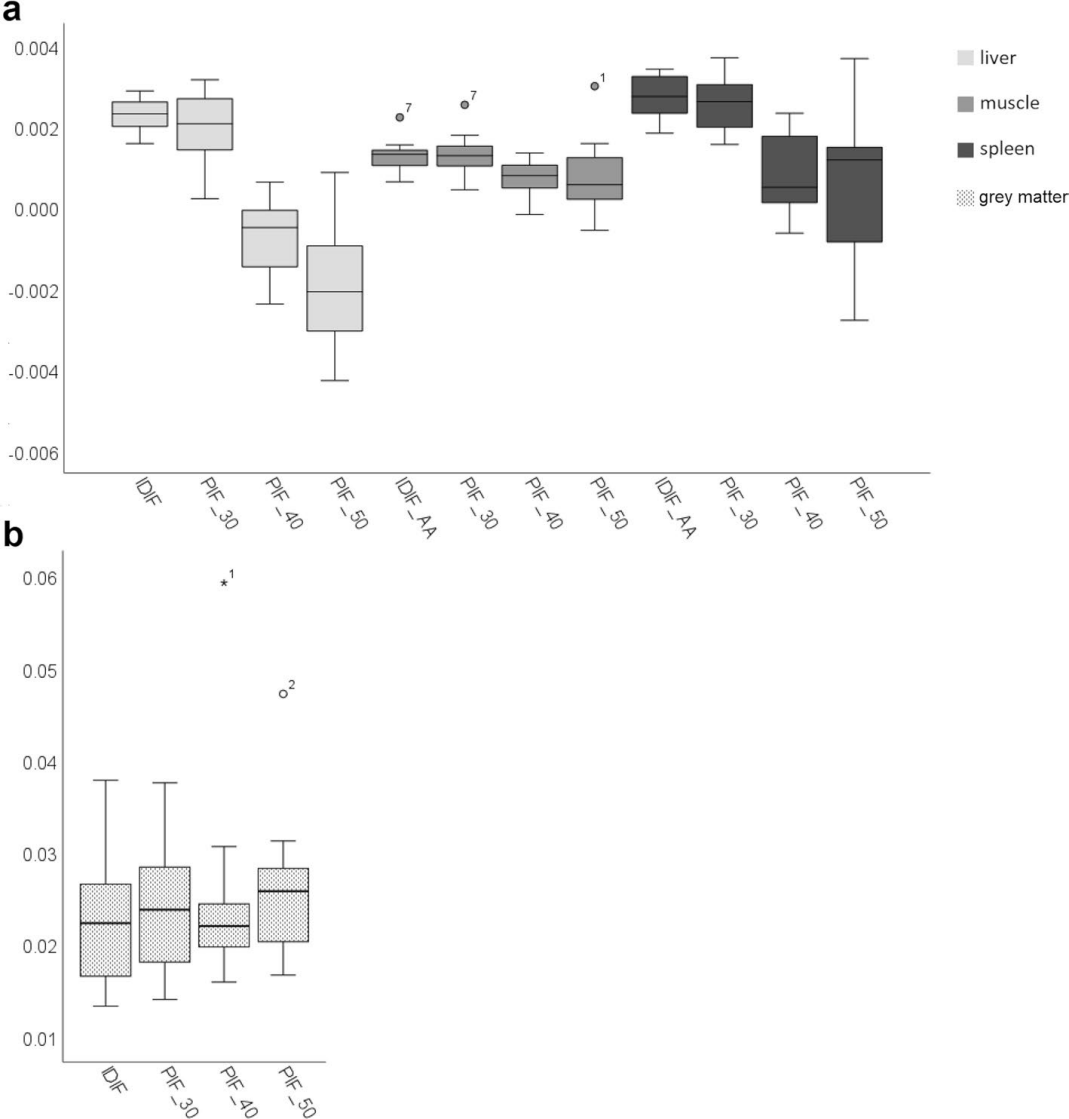
Healthy tissue *K*_*i*_ obtained using the IDIF compared with *K*_*i*_ obtained with the PIF scaled to the IDIF at scan time interval 30–60 min p.i. (PIF_30), 40–60 min p.i. (PIF_40), and 50–60 min p.i. (PIF_50). Mild outliers are marked with a circle and are data points that fall outside ${\text{quartile}}1\,\left( {Q1} \right) - 1.5*{\text{interquartile}}\,{\text{range}} \,\left( {{\text{IQR}}} \right)$ or ${\text{Q}}3 + 1.5 *{\text{ IQR}}$. Extreme outliers are marked with an asterisk and fall outside ${\text{Q}}1 - 3 *{\text{ IQR}}$ or ${\text{Q}}3 + 3 *{\text{ IQR}}$. For readability, axes were scaled differently for liver, spleen, and muscle *K*_*i*_ (**a**) with respect to grey matter *K*_*i*_ (**b**)

Comparison of healthy tissue *K*_*i*_ of liver, muscle tissue, spleen and grey matter obtained using the IDIF versus the corresponding scaled PIF at various shortened scan time intervals p.i. are shown in Fig. 6. ANOVA repeated measures show significant differences in liver *K*_*i*_ obtained using the IDIF and the PIF at 40–60 min (confidence interval (CI) (0.002–0.004), *P* < 0.001) and at 50–60 min scan time interval p.i. (CI (0.003–0.00, *P* < 0.001). Significant differences were also found between liver *K*_*i*_ obtained using the PIF at 30–60 min p.i. scan time interval and the PIF at shortened time intervals of 40–60 min (CI (0.002–0.003),* P* < 0.001) and 50–60 min (CI (0.003–0.005), *P* < 0.001). For muscle tissue, ANOVA repeated measures showed significant differences in *K*_*i*_ obtained using IDIF and the PIF scaled to the IDIF at 40–60 min p.i. (CI (0.000–0.001),* P* < 0.001) and between *K*_*i*_ obtained using the PIF at 30–60 min versus 40–60 min (CI (0.000–0.001), *P* < 0.001. With regard to the spleen, significant differences were found in *K*_*i*_ obtained using the IDIF and the corresponding shortened PIF scaled to 40–60 min p.i. (CI (0.001–0.003), *P* < 0.001) and scaled to 50–60 min p.i. (CI (0.001–0.004), *P* < 0.001). Analysis of grey matter *K*_*i*_ showed a significant difference in *K*_*i*_ obtained using the IDIF and the PIF scaled to 30–60 min p.i. (CI (− 0.002–0.001), *P* = 0.001).

## Discussion

Over the last decade, improvements in detector technology [3, 32] and higher demands for clinical application have led to the development and recent introduction of LAFOV PET systems [32, 33]. Apart from the Vision Quadra PET/CT, other LAFOV PET systems that have been introduced recently are the uEXPLORER (United Imaging Healthcare, Houston, TX, USA) with a LAFOV of 194 cm [24, 34] and the PennPET Explorer (University of Pennsylvania, Philadelphia, PA, USA) with a LAFOV of 64 cm [35, 36]. Due to the large axial FOV of these systems, access to arteries for arterial sampling to obtain the arterial IF to allow full quantitative kinetic analysis is challenging. Because of the substantial increase in sensitivity with regard to conventional PET systems (with a more limited axial FOV) and the corresponding lower noise levels, LAFOV systems allow for non-invasive whole body dynamic ^18^F-FDG Patlak imaging methods using an IDIF (validated against the gold standard arterial blood sampling in [37]). However, whereas improvements in PET technology allow whole body parametric imaging, these also enable acquisition of whole body static images at substantially reduced scan durations for increased patient throughput. Hence, the assessment of non-invasive whole body ^18^F-FDG Patlak imaging using a PIF for acquisition at shortened scan time intervals to reduce scan time for whole body parametric imaging as conducted in this study, will be a useful and compatible method to apply with these LAFOV PET systems.

The current study shows that non-invasive whole body ^18^F-FDG Patlak imaging to be feasible using a representative PIF scaled to the IDIF obtained from the AA at 30–60 min p.i. An even shorter scan time interval of 40–60 min p.i. may also be a good compromise between overall scan duration and quantitative robustness of tumour *K*_*i*_ values, although it is associated with an increase in noise and a slight deterioration in image quality. In the present study, *K*_*i*_ estimates deviated marginally (7.38%) when using the PIF scaled to the IDIF at 30–60 min p.i. with respect to using the IDIF itself. The observed deviation agrees well with those as published before [23]. An accuracy level of 7.38% is well within the tumour SUV quantification repeatability levels of 10–15% [38–40]. Although Viswanath et al. also studied an abbreviated whole body dynamic ^18^F-FDG imaging protocol for the Vision Quadra, they recommend 20 min total scan time subdivided into 10–15 min p.i. and 5 min at 60 min p.i., which could be less pragmatic for routine clinical implementation. For example, the dual time point approach would require each subject to be positioned on the system twice, which may add to dead time in the use of the system, requires additional patient handling, and consequently hamper a busy clinical workflow. In addition, a second CT acquisition is required which increases total radiation exposure. Finally, both image datasets need to be coregistered carefully, possibly requiring non-rigid transformations.

When using the PIF scaled to the IDIF at 40–60 min, *K*_*i*_ bias decreased, but the SD in *K*_*i*_ obtained from the liver increased, indicating a trade-off between image quality (Fig. 1) and shorter scan duration at the expense of increased noise. *K*_*i*_ bias increases to > 24% when using even shorter scan duration, i.e. 50–60 min (see Figs. 3 and 4), which is no longer clinically acceptable. In addition, this shorter scan interval was associated with substantial increase in noise. Moreover, for some organs ^18^F-FDG may show reversible kinetics, such as liver and spleen. The latter explains the change or decrease in Ki with later t* values and for these organs, generalised Patlak analysis or spectral analysis is more appropriate [12, 13, 17, 41]. For tumours other than hepatocellular carcinoma [42], this phenomenon is unseen and use of Patlak with t* = 30 or 40 min, and thus analysis time intervals of 30 to 60 min or 40 to 60 min, did not introduce bias while maintaining sufficient precision (Figs. 3 and 4).

Benefits of incorporating shorter scan times are not only patient throughput, but also patient comfort. A short 20 to 30 min (dynamic) scan protocol at the interval of 40–60 or 30–60 min p.i. is more likely to be tolerated with respect to the standard (at least) 65 min that is necessary for a full whole body dynamic Patlak acquisition. In addition, parametric images obtained using 20 or 30 min scan duration are less prone to motion artefacts [43, 44].

With regard to clinical advantages of whole body dynamic Patlak imaging over conventional static scans, parametric images can provide complementary information to standard SUV images, or rather filter information by deleting intravascular contributions to the PET signal, enabling easier detection and classification of small ^18^F-FDG avid lesions, particularly in high background uptake regions, such as the liver [43, 45]. However, even with the possibility to reduce scan duration to 20 min, including the low dose CT and patient positioning, examination time will increase up to 30 min. LAFOV systems allow standard whole body static scan durations of less than 2 min [36, 46, 47], including patient positioning and CT this could lead to a total examination time of $\sim$ 10 to 15 min, which is a factor 3 to 2 less with respect to the shortest feasible whole body Patlak acquisition procedure. Based on these findings and the specific aims regarding patient throughput at different PET centres, dynamic whole body Patlak imaging may not be suitable for all patient studies; for diagnostics and staging, a simple static scan would do. However, for select patient groups, additional information to more accurately monitor treatment response may be required, especially when comparing to a baseline scan. In those cases ^18^F-FDG blood clearance changes may affect SUV based quantification [9, 30, 45, 48, 49].

### Limitations

We did not validate the IDIF against the gold standard, arterial sampling for determining the arterial input curve. However, using the AA for the IDIF allowed us to define a large VOI to provide us data with good statistical properties without risk of spill-over from potentially high uptake tissues like the myocardium [28]. Furthermore, the standard Patlak model was used in the current study, assuming an irreversible 2-tissue compartment tracer kinetic model. However, several studies have reported non-negligible ^18^F-FDG uptake dephosphorylation in some healthy tissue organs as well as in hepatocellular carcinoma [50], causing the standard Patlak model to underestimate *K*_*i*_ [13]; in these cases the generalised Patlak model is recommended for a more accurate model fit [13, 41]. Nonetheless, in [41] it was shown that in healthy tissue organs such as the liver, the *R*^2^ of the standard Patlak plot fit fell between 0.85 and 0.95 and an acceptable linear trend was seen. Reversibility of ^18^F-FDG tracer uptake in lung carcinoma or lymphoma has never been shown, and therefore, we assume the standard irreversible 2-tissue compartment model to be valid to use in the current study. Nonetheless, using the generalised Patlak could have resulted in more accurate results for organs and regions showing reversible kinetics which would be of interest to explore in future whole body dynamic studies.

## Conclusion

Our study shows that parametric whole body ^18^F-FDG Patlak *K*_*i*_ images can be obtained non-invasively (e.g. without the need for blood sampling) using a Vision Quadra PET/CT system. In addition, using a PIF scaled to the IDIF allows for a factor 2 to 3 reduction in scan time, from 65 min to a scan time interval of 30–60 or 40–60 min p.i., without substantial loss of accuracy (7.38% bias at most) and precision (image quality and noise interference).

## Supplementary Information

**Additional file 1: Fig. S1.** Plot of population-averaged input function. The *y*-axis is in logarithmic scale. **Fig. S2.** Bland–Altman plot of lesion *K*_*i*_ differences obtained with the IDIF minus the PIF scaled to the scan time interval at 30–60 min p.i. (PIF_30) (A), and the PIF scaled to the IDIF at shorter scan time intervals p.i. (PIF_40 and PIF_50) (B and C). For readability, the scales have been adjusted per subfigure.

## Data Availability

The data that support the findings of this study are available on request from the corresponding author JS. The data are not publicly available because they contain information that could compromise research participant privacy/consent.
