# Supplementary material for: Shortened duration whole body 18F-FDG PET Patlak imaging on the Biograph Vision Quadra PET/CT using a population-averaged input function
Source: EJNMMI Phys. 2022 Oct 29;9:74. doi: 10.1186/s40658-022-00504-9 (PMC9618000; doi:10.1186/s40658-022-00504-9)
Supplement: Supplementary file 1 — Additional file 1: Fig. S1. Plot of population-averaged input function. The y-axis is in logarithmic scale. Fig. S2. Bland–Altman plot of lesion Ki differences obtained with the IDIF minus the PIF scaled to the scan time interval at 30–60 min p.i. (PIF_30) (A), and the PIF scaled to the IDIF at shorter scan time intervals p.i. (PIF_40 and PIF_50) (B and C). For readability, the scales have been adjusted per subfigure. [file 40658_2022_504_MOESM1_ESM.docx]

**
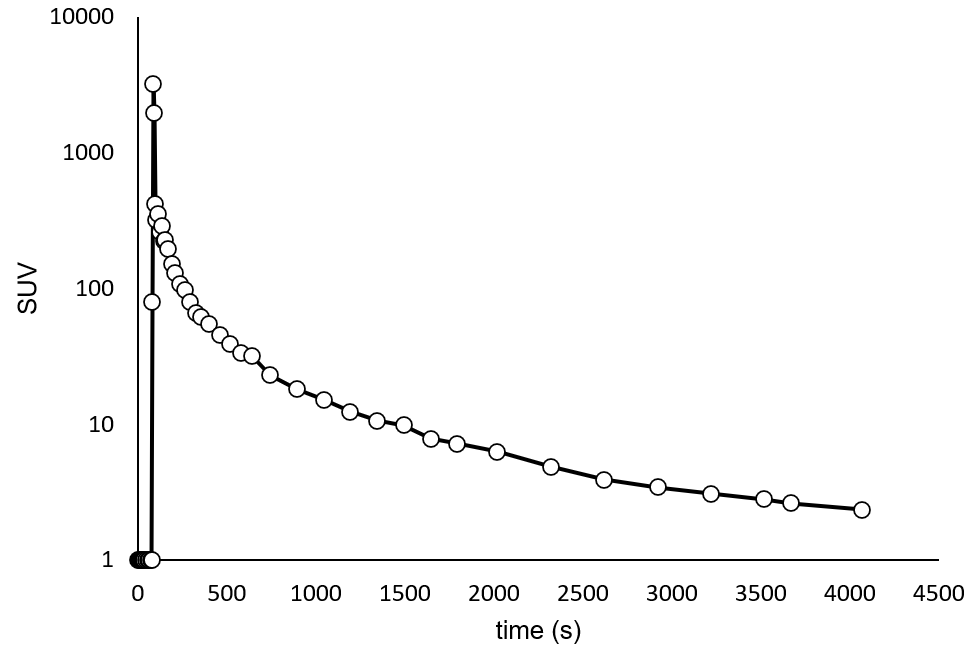
SUPPLEMENTAL DATA**

**Fig. S1** Plot of population-averaged input function. The y-axis is in logarithmic scale.


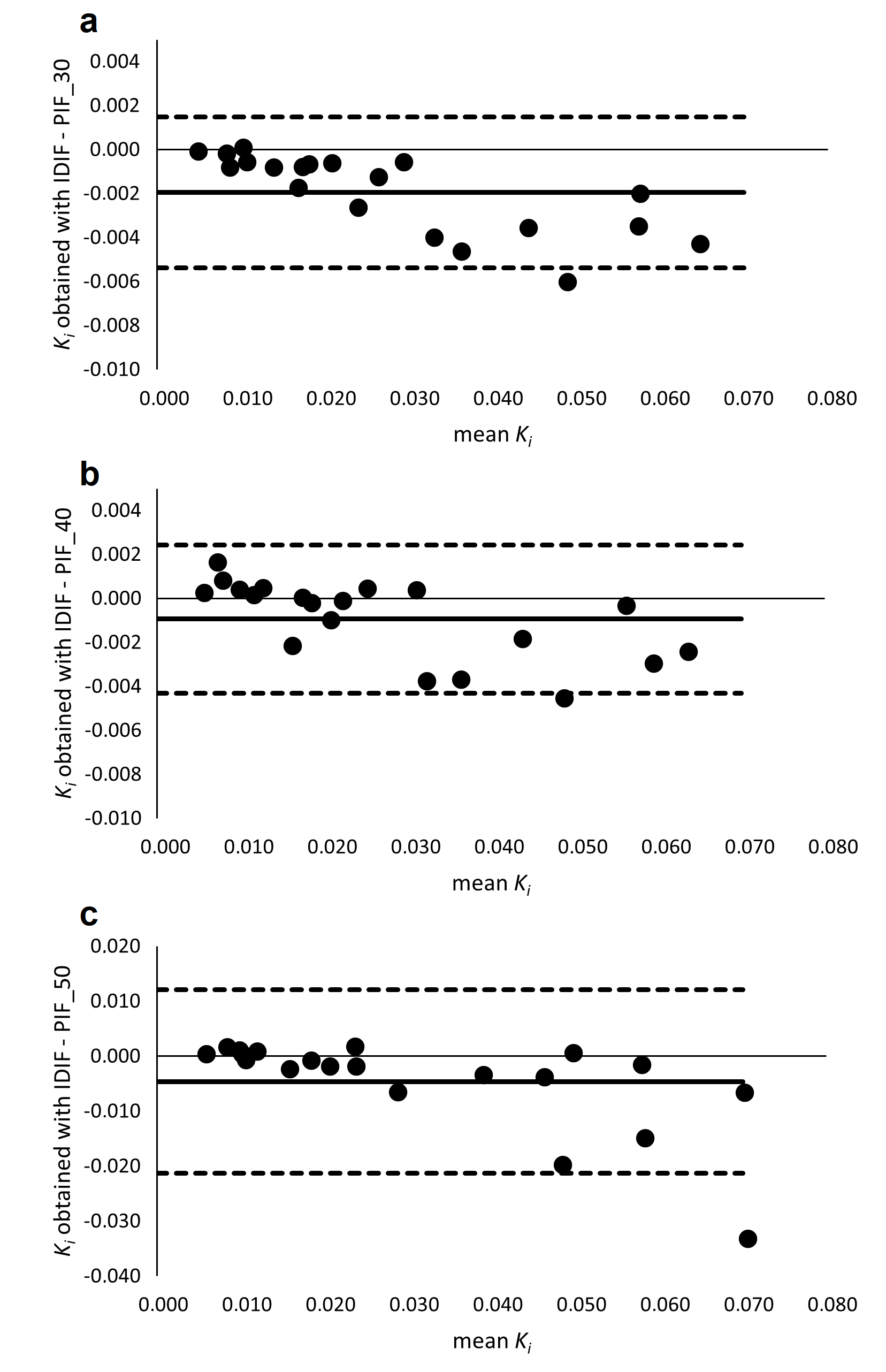


**Fig. S2** Bland-Altman plot of lesion K_i_ differences obtained with the IDIF minus the PIF scaled to the scan time interval at 30-60 min p.i. (PIF_30) (A), and the PIF scaled to the IDIF at shorter scan time intervals p.i. (PIF_40 and PIF_50) (B and C). For readability, the scales have been adjusted per subfigure.
